# Field and controlled environment measurements show strong seasonal acclimation in photosynthesis and respiration potential in boreal Scots pine

**DOI:** 10.3389/fpls.2014.00717

**Published:** 2014-12-12

**Authors:** Pasi Kolari, Tommy Chan, Albert Porcar-Castell, Jaana Bäck, Eero Nikinmaa, Eija Juurola

**Affiliations:** ^1^Department of Physics, University of HelsinkiHelsinki, Finland; ^2^Department of Forest Sciences, University of HelsinkiHelsinki, Finland

**Keywords:** gas exchange, chlorophyll fluorescence, annual cycle, *P*_max_

## Abstract

Understanding the seasonality of photosynthesis in boreal evergreen trees and its control by the environment requires separation of the instantaneous and slow responses, as well as the dynamics of light reactions, carbon reactions, and respiration. We determined the seasonality of photosynthetic light response and respiration parameters of Scots pine (*Pinus sylvestris* L.) in the field in southern Finland and in controlled laboratory conditions. CO_2_ exchange and chlorophyll fluorescence were measured in the field using a continuously operated automated chamber setup and fluorescence monitoring systems. We also carried out monthly measurements of photosynthetic light, CO_2_ and temperature responses in standard conditions with a portable IRGA and fluorometer instrument. The field and response measurements indicated strong seasonal variability in the state of the photosynthetic machinery with a deep downregulation during winter. Despite the downregulation, the photosynthetic machinery retained a significant capacity during winter, which was not visible in the field measurements. Light-saturated photosynthesis (*P*_sat_) and the initial slope of the photosynthetic light response (α) obtained in standard conditions were up to 20% of their respective summertime values. Respiration also showed seasonal acclimation with peak values of respiration in standard temperature in spring and decline in autumn. Spring recovery of all photosynthetic parameters could be predicted with temperature history. On the other hand, the operating quantum yield of photosystem II and the initial slope of photosynthetic light response stayed almost at the summertime level until late autumn while at the same time *P*_sat_ decreased following the prevailing temperature. Comparison of photosynthetic parameters with the environmental drivers suggests that light and minimum temperature are also decisive factors in the seasonal acclimation of photosynthesis in boreal evergreen trees.

## Introduction

The boreal zone is characterized by the large amplitude and dynamic annual pattern of light and temperature. These dynamics are known to control the strong seasonal variation in photosynthetic capacity of boreal evergreen foliage. Yet, the understanding on these controls is limited due to the lack of reliable and informative long-term measurements and meaningful analysis of empirical data.

Photosynthesis is controlled by light and temperature at two different time scales and subprocesses. Instantaneous changes in radiation affect the rate of photon capture by the light reactions of photosynthesis, and contribute also to the activation/deactivation of CO_2_-binding enzyme Rubisco, and stomatal control (e.g., Pearcy, 1990). Instantaneous changes in temperature modulate all enzymatic steps of photosynthesis. Seasonal changes in light and temperature also control the dynamics of photosynthetic capacity, i.e., the potential CO_2_ uptake under optimal conditions, where the capacity of both light and carbon reactions of photosynthesis is up- or downregulated by environmental and plant-level physiological cues via different signaling pathways (Öquist and Hüner, 2003; Pfannschmidt, 2003; Ensminger et al., 2006). The instantaneous responses of photosynthesis and leaf respiration to the environmental drivers change seasonally within the range determined by the current state of the photosynthetic light and carbon reactions and the whole leaf physiology. Accordingly, understanding the seasonality of photosynthesis and its control by the environment requires separation of the instantaneous and slow responses, as well as the dynamics of light reactions, carbon reactions, and respiration.

The complete picture how the state of the photosynthetic machinery varies seasonally is somewhat unclear due to different approaches to how the state is defined as well as different measurement setups and conditions in the various empirical studies. The state can be defined as the capacity or maximum process rate (such as light-saturated photosynthesis), process rate in standard conditions (respiration in standard temperature), or as the efficiency (the slope of photosynthetic rate as a function of light or CO_2_) of a physiological process. The state is sometimes described with one lumped parameter (e.g., maximum photosynthetic rate *P*_max_) without explicitly addressing the capacity of photosynthetic light and carbon reactions. Often the other state parameters are considered constant, e.g., constant *J*_max_/*V*_cmax_ ratio or fixed temperature response of respiration.

The seasonality of photosynthetic capacity has been linked to temperature (Pelkonen and Hari, 1980; Mäkelä et al., 2004). There is also evidence of the effect of photoperiod on downregulation of photosynthetic light reactions in autumn (Busch et al., 2007), and light intensity and temperature on the recovery during spring (Ensminger et al., 2004; Porcar-Castell et al., 2008a). Mäkelä et al. (2004) introduced a theoretical variable, state of acclimation (*S*), which describes the state of acclimation of the photosynthetic apparatus in temperature units. The seasonality of photosynthetic capacity in boreal Scots pine (*Pinus sylvestris* L.) was successfully predicted as slow response toward prevailing temperature (Mäkelä et al., 2004; Kolari et al., 2009; Gea-Izquierdo et al., 2010). However, in those studies the instantaneous response to temperature was largely omitted and the modeling approach reflected parameters estimated at prevailing temperature rather than the full capacity.

Several other recent studies on the seasonality of photosynthesis and respiration were also based on data collected in prevailing conditions in the field (Kolari et al., 2007; Ow et al., 2010; Linkosalo et al., 2014). Continuous measurements in the field give limited or biased information on the potential photosynthetic capacity that the plant would have in optimal conditions, which would indicate better the current physiological state. Furthermore, determining the instantaneous photosynthetic responses to different environmental drivers, i.e., light, temperature, air humidity or VPD, from continuous field measurements is uncertain. The drivers are mutually correlated and the range of short-term variability can be narrow, for instance, low light and small diurnal variation of temperature in boreal autumn. Determination of the underlying state of the leaf requires measurements under a wide range of conditions and breaking the correlations of the drivers, something that becomes difficult in the field especially during boreal winters. Accordingly, disentangling the instantaneous responses to light or temperature from the changes in the state in the observed seasonal pattern in photosynthesis remains a challenge. Studying the responses to the environment is further complicated by the tree-level effects on instantaneous photosynthetic rate (e.g., water supply, availability of nutrients, sink control) that are embedded in the responses of photosynthetic rate to the environment (Nikinmaa et al., 2013).

Permanent shoot chamber enclosure systems coupled to infra-red gas analyzers (IRGA) can be used for monitoring net CO_2_ exchange over long periods of time in the field and, to some extent, for determining physiological parameters (Kolari et al., 2007, 2009). Photosynthesis and respiration can be derived from these measurements at time resolution of a few minutes. Alternatively, chlorophyll fluorescence measurements can be also used for long-term monitoring of photosynthesis, in terms of electron transport, at a similar temporal resolution (Porcar-Castell et al., 2008b; Porcar-Castell, 2011). Portable systems capable of measuring simultaneously gas exchange and chlorophyll fluorescence are currently widely used in point measurements of leaf-level photosynthetic parameters. These systems are very versatile and allow adjustment of the incoming light, temperature, air humidity and CO_2_ concentration in the measuring chamber. Combining continuous measurements on intact shoots in the field and intermittent measurements of excised shoots in controlled conditions can give more information about the dynamics and responses of photosynthesis and respiration to different drivers, and thus about the state of the photosynthetic machinery. Tree-scale factors such as varying water status and photosynthate transport capacity can also be partly eliminated when studying excised shoots.

In this study we assess how photosynthetic and respiration parameters observed in the field and in standard near-optimal conditions are related to each other in Scots pine growing in the boreal zone. This entails determining the seasonality of CO_2_ exchange and chlorophyll fluorescence in the field using continuous measurements with automated chambers and fluorescence monitoring systems and in controlled conditions with periodically repeated measurements of photosynthetic light, CO_2_ and temperature responses with a portable IRGA system coupled with a fluorometer. We separately determine the seasonality of photosynthetic light and carbon reaction parameters and respiration in standard conditions and quantify to what extent the observed dynamics in photosynthesis and respiration in the field are instantaneous responses and to what extent they reflect slow changes in the state of the leaf physiology. Finally, we discuss how accurate information about the state can be obtained from field measurements and evaluate how the model of slow temperature acclimation (Mäkelä et al., 2004), previously tested against field monitoring data, can explain the observed seasonal patterns in the photosynthetic light and carbon reaction parameters determined in standard conditions.

## Materials and methods

### Study site

Dynamics of photosynthesis in Scots pine (*Pinus sylvestris L*.) was studied at Helsinki University SMEAR II (Station for Measuring Forest Ecosystem-Atmosphere Relations) field station in Hyytiälä, southern Finland (61°51′N, 24°17′E, 180 m a.s.l.). The station is situated in a lightly managed Scots pine stand established in 1962. The mean annual precipitation and temperature at the site were 711 mm and 3.5°C, respectively, for 1980–2010 (Pirinen et al., 2012). The mean daily, mean daily maximum, and mean daily minimum temperatures were −7.2, −4.4, and −10.8°C in January, and 16.0, 21.6, and 10.8°C in July, respectively.

### Measurements of photosynthesis, leaf respiration and fluorescence

#### Continuous monitoring

Continuous gas exchange measurements on pine shoots during years 2010–2011 were analyzed in this study. The instrumentation consisted of chambers, sample tubing, gas analyzers and a control unit operating the system automatically. The chambers were made of acrylic plastic with 1 dm^3^ volume. The chambers were open most of the time exposing the chamber interior to the ambient conditions. For measuring fluxes, the chambers were closed intermittently for 1 min. Measurements of CO_2_ and water vapor fluxes and concentrations, air temperature inside the chambers and photosynthetically active radiation (PAR) outside of the chambers were done 50–80 times a day. During the chamber closure, gas concentrations and environmental variables were recorded every 5 s. The flux calculation was based on the detection of the gas concentration change in the chambers during the closure (Hari et al., 1999). Altimir et al. (2002) described the instrumentation in more detail.

The studied shoots were located at the top of the canopy in two trees. The chambers were installed on the shoots so that they accommodated one age class of needles. The terminal buds were removed prior to chamber installation to prevent new growth inside the chambers. Three chambers were in use simultaneously from 2 March 2010 until 10 January 2011, in other times there were two chambers. After completing the measurements on the shoots, the dimensions of the needles in each shoot were measured and their surface area was calculated using the equation from Tiren (1927) and divided by 3 to obtain projected area.

Continuous measurements of chlorophyll fluorescence were conducted using a Monitoring PAM fluorometer system (Heinz Walz, GmbH, Germany) consisting of several independent measuring heads (Porcar-Castell et al., 2008b; Porcar-Castell, 2011). The number of parallel needle samples was most of the time three or four. Three to four pairs of needles were clipped in each measuring head and the prevailing (*F*′) and maximal fluorescence (*F*′_m_) were measured every 15, 30, or 60 min using the saturating pulse technique (Schreiber et al., 1986). Measuring frequency was adjusted during the season to minimize pulse-induced long-term photoinhibition, using lower frequencies during winter and nights. The duration of the saturating light pulse was 0.8 s and the intensity at the leaf-surface was >4000 μmol m^−2^ s^−1^. The data was used to estimate the operating quantum yield of photochemistry in photosystem II (PSII) (Genty et al., 1989), as

(1) $$\phi_{\text{PSII}}\;=\;\frac{{F^{\prime }}_{\text{m}}-F^{\prime }}{{F^{\prime }}_{\text{m}}}$$

In this study we present the daily maximum Φ_PSII_. This yield was obtained during nighttime and it is equivalent to the parameter *F*_v_/*F*_m_ in dark acclimated samples (Kitajima and Butler, 1975; Maxwell and Johnson, 2000). We denote the parameter *F*_v_/*F*_m_ to distinguish it from the yield determined in controlled conditions.

#### Response measurements

Six overstory trees (height about 15 m) were randomly selected for the response measurements. The measurements were started in February 2010 and continued approximately once a month through the following winter until December 2011, in total 22 times. At each measuring point a branch was cut from the upper canopy of each tree, placed under water and brought to the laboratory. Branches were subsequently re-cut under water. Time from the transfer to laboratory conditions to starting the measurements varied from 30 min to 5 h. Each monthly measurement was collectively sampled over a period of 3–4 days.

The CO_2_ exchange rates and operating quantum yields of photosystem II were measured with a portable IRGA equipped with an integrated fluorometer (Walz GFS-3000, Heinz Walz, Germany). For each branch, four fascicles, totaling eight needles were placed in the measuring cuvette. Cohorts from 2009 to 2010 were used during 2010 and 2011 measurements, respectively.

Three different response measurement sequences were performed: (i) light response at ambient temperature and ambient CO_2_ concentration, (ii) light response at standard temperature of 18°C and ambient CO_2_ concentration, and (iii) CO_2_ response at standard temperature of 18°C and saturating light (1300 μmol m^−2^ s^−1^). Before the start of the full measurement sequence the needles were treated with an initial 15–20 min stabilization period in conditions close to ambient field temperature (but not below −1°C) and 600 μmol m^−2^ s^−1^ PAR. After the first light response sequence the needles were allowed to acclimate for 30–45 min when temperature was changed from ambient to standard conditions. The increase in temperature was performed in two steps, if the temperature difference was large. Similarly, the CO_2_ sequence had an initial stabilization period of 15–20 min.

A comprehensive response of leaf photosynthesis to light was produced by stepwise changes in light from the initial 600 μmol m^−2^ s^−1^ sequentially to 400, 100, 50, 25, 0, 600, 900, 1200, and 1700 μmol m^−2^ s^−1^, with a stabilization period of 150 s between each step plus seven successive measurements at 5-s intervals at each light level. The CO_2_ response (*A*-*C*_i_), was measured at 18°C at constant 1300 μmol m^−2^ s^−1^ PAR with stepwise changes in CO_2_ concentrations, starting from 380 μmol mol^−1^ and followed by 200, 100, 50, 25, 380, 600, 800, and 1200 μmol mol^−1^. Operating quantum yield of photosystem II (Φ_PSII_) was determined at the end of each light and CO_2_ step using a saturating pulse of 0.8 s duration and >4000 μmol intensity, following the same method as in the Monitoring PAM system described above.

Temperature responses at 800 μmol m^−2^ s^−1^ PAR were measured three times in 2011. The sequence was started with stabilization at 16°C, temperature was then decreased to 8°C in 4°C steps and finally increased to 24°C in 4°C steps. Time between each step was 10 min. Net CO_2_ exchange and photosynthesis in the temperature response measurements are denoted *A*_800_ and *P*_800_, respectively.

During the measurements the flow rate through the cuvette was 650 μmol s^−1^, relative humidity was kept between 55 and 70%, and CO_2_ concentration during the light and temperature response sequences was stabilized to 380 μmol mol^−1^. The conditions were controlled with the GFS-3000.

Leakage from or into the cuvette may occur when the difference in CO_2_ concentration between the cuvette and outside air is large. The effect of leakage on the observed CO_2_ exchange was quantified by recording the standard gas exchange measurement sequence with an empty cuvette and estimating CO_2_ exchange as linear function of concentration difference between the cuvette and outside air. All the CO_2_ exchange data were corrected by this method.

After the measurements, the needles were photographed and their projected area calculated with image analysis software (ImageAnalyzer, Dr. Martti Perämäki). The needles were then collected to measure their fresh weight, length, thickness and width, and dried at 105°C for 24 h to get the dry weight.

### Models of photosynthesis and respiration

#### Simple light response of photosynthesis

Net CO_2_ exchange of leaves (*A*) consists of photosynthetic CO_2_ uptake (*P*) and CO_2_ efflux from dark respiration (*R*_d_). It can be expressed as a saturating function of light:

(2) $$A=\frac{1}{2\theta }\left[\alpha I+P_{\text{sat}}-\sqrt{{\left(\alpha I+P_{\text{sat}}\right)}^{2}-4\theta \alpha IP_{\text{sat}}}\right]\;-R_{\text{d}}$$

We estimated daily light-saturated photosynthesis *P*_sat_, the initial slope of the light response α_f_ and dark respiration *R*_d_ from the field CO_2_ exchange measurements. From the response measurements we determined the initial slope α_s_, *R*_d_ and photosynthetic rate measured in 1200 μmol m^−2^ s^−1^ PAR (*P*_1200_) that represented *P*_sat_. This light intensity was chosen, because the response measurements were performed throughout the year and during winter higher light intensities resulted in photoinhibition and decreased photosynthesis. We fixed θ to 1 (Blackman curve) to obtain more robust estimates for the other parameters. Respiration in the light was assumed to be the same as in the darkness.

#### Biochemical model of photosynthesis

The maximum electron transport rate (*J*_max_), maximum carboxylation rate (*V*_cmax_) and apparent dark respiration during the day (*R*_d_) were determined from the *A-I* and *A*-*C*_i_ response data set by fitting the biochemical photosynthesis Farquhar et al. (1980) model:

(3) $$A=\min\left\{W_{\text{c}},W_{\text{j}}\right\}-R_{\text{d}}$$

where *W*_j_ is light-limited photosynthesis

(4) $$W_{\text{j}}\;=\;\frac{J\left(C_{\text{i}}-\Gamma^{*}\right)}{4C_{\text{i}}+8\Gamma^{*}}$$

*J* is electron transport rate

(5) $$J=\frac{\alpha I+J_{\max}\;-\;\sqrt{{\left(\alpha I+J_{\max}\right)}^{2}-4\alpha \theta IJ_{\max}}}{2\theta }$$

and *W*_c_ is carbon-limited photosynthesis

(6) $$W_{\text{c}}\;=\;\frac{V_{\text{c}\max}\left(C_{\text{i}}-\Gamma^{*}\right)}{C_{\text{i}}+K_{\text{C}}\left(1+O_{2}/K_{\text{O}}\right)}$$

*V*_cmax_ and *J*_max_ were estimated from the low (*C*_i_ < 350 μmol mol^−1^) and high (*C*_i_ > 350 μmol mol^−1^) *C*_i_ regions of the *A*-*C*_i_ responses, respectively (Wullschleger, 1993). The values of *K*_c_, *K*_o_, and Γ^*^ were taken from literature (Farquhar et al., 1980). In addition to the widely used parameters *V*_cmax_ and *J*_max_ we also estimated the initial slope of the *A*-*C*_i_ curve which requires less assumptions than *V*_cmax_ on the limitations and the values of the other model parameters.

#### Temperature responses of photosynthesis and respiration

The instantaneous temperature responses of photosynthetic model parameters were analyzed with a function that addresses the low-temperature inhibition of photosynthesis (Collatz et al., 1992). The rate of photosynthesis *P* at leaf temperature *T* is

(7) $$P(T)\;=\;P\left(T_{\text{ref}}\right)\frac{e^{s\left(T-T_{\text{ref}}\right)}}{\left(1+e^{s_{1}\left(T-T_{1}\right)}\right)\left(1+e^{s_{2}\left(T_{2}-T\right)}\right)}$$

where *P*(*T*_ref_) is the photosynthetic rate at reference temperature *T*_ref_, *s* determines the slope of the response function at intermediate temperatures, *s*_1_ and *T*_1_ determine the high-temperature inhibition and *s*_2_ and *T*_2_ the low-temperature inhibition. In this study *T*_ref_ was 18°C, the standard temperature in the response measurements.

Seasonality of photosynthetic capacity was previously described as slow acclimation to prevailing temperature (Mäkelä et al., 2004). The state of acclimation *S* is defined as a function of leaf temperature *T* and time constant τ

(8) $$\frac{dS}{dt}\;=\;\frac{T-S}{\tau }$$

*S* can be thought as the temperature that the photosynthetic machinery is acclimated to and it is expressed in temperature units. We use as reference to the seasonal patterns of the photosynthetic parameters the sigmoid relationship between photosynthetic capacity β and *S* estimated by Kolari et al. (2007):

(9) $$\beta \;=\;\frac{\beta_{\max}}{1+e^{b\left(S-T_{\text{S}}\right)}}$$

where β_max_ is the maximum summertime photosynthetic capacity and *b* and *T*_S_ are empirically determined parameters. Originally the capacity parameter β determined the rate of light-saturated photosynthesis per unit *C*_i_ in the model of optimal stomatal control (Hari et al., 1986). In other words, β = *P*_sat_/*C*_i_. We also test this model formulation for other photosynthetic parameters.

Respiration was analyzed with the commonly used *Q*_10_ Equation

(10) $$R\;=\;R_{0}Q_{10}^{T/10}$$

where *Q*_10_ is the temperature sensitivity and *R*_0_ respiration in 0°C.

## Results

### Seasonal patterns of environmental drivers and CO_2_ fluxes

**Figure 1:**
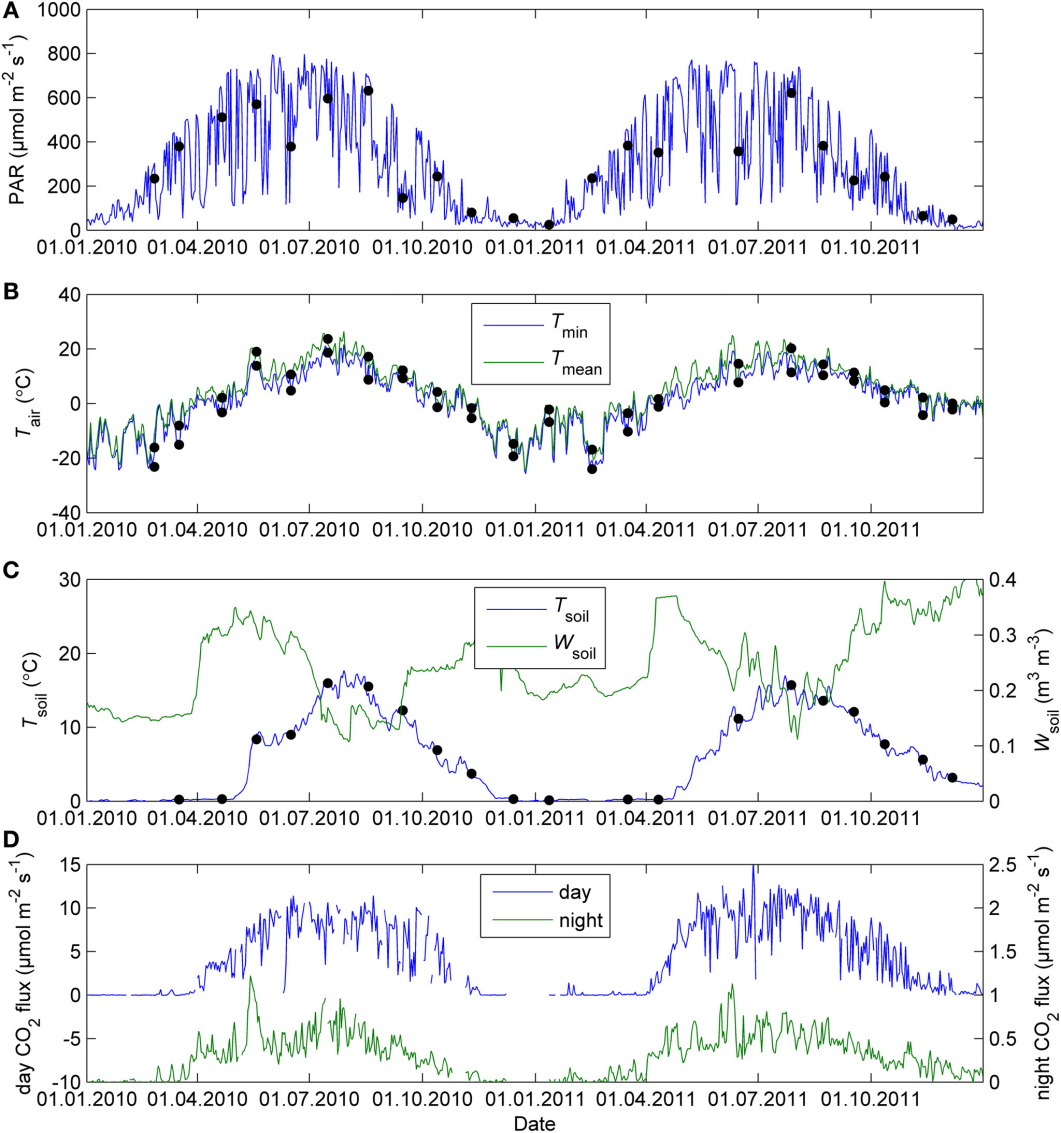
**Daily mean PAR (A), minimum and mean air temperature (B), soil volumetric water content and soil temperature (C), and midday and midnight CO_2_ exchange of Scots pine shoots in the field (D) in Hyytiälä in 2010–2011**. The dots indicate 5-day mean PAR, mean and minimum ambient temperature and soil temperature for the response measurement campaigns. The CO_2_ fluxes were averaged daily between 11:00 and 14:00 (day) and 23:00 and 02:00 (night) local normal time.

The study period was characterized by the cold winter and hot and dry midsummer of 2010, more typical winter 2010–2011 and summer of 2011 and the warm end of year 2011. The seasonal patterns of environmental conditions during the study are shown in Figure 1. Figure 1D shows the seasonal courses of noon and midnight CO_2_ fluxes in the field.

### Seasonality of photosynthetic and respiration parameters

**Figure 2:**
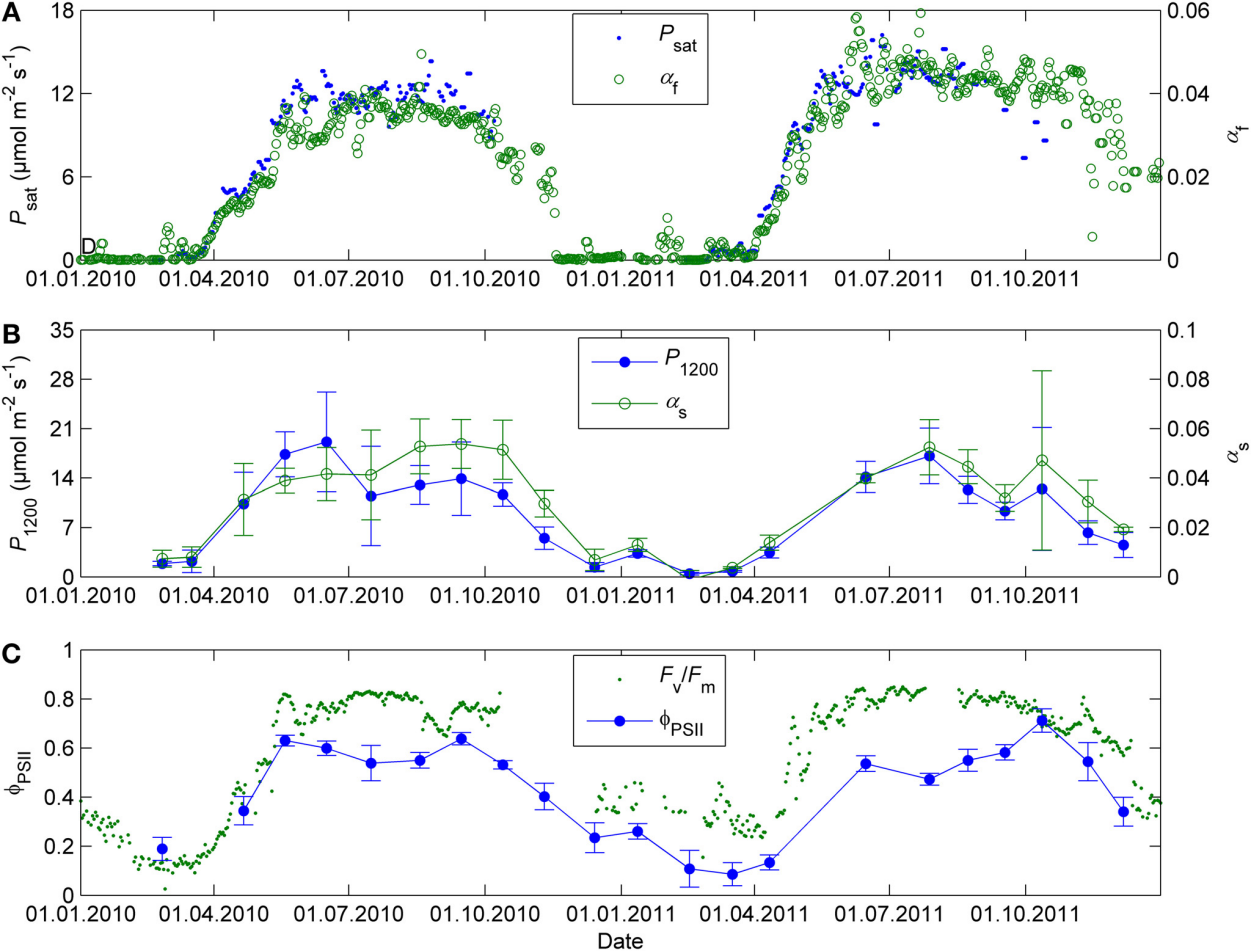
**Daily initial slope of photosynthetic light response α_f_ and light-saturated photosynthesis *P*_sat_ in 2010–2011 estimated from the continuous gas exchange data in the field (A), corresponding parameters α_s_ and *P*_1200_ determined using response mesurements in controlled conditions at 18°C and 380 ppm (B), and fluorescence yield in the field conditions (*F*_v_/*F*_m_) and in controlled conditions of 50 μmol m^−2^ s^−1^ PAR, 18°C and 380 ppm (Φ_PSII_) (C)**. The field gas exchange parameters **α**_f_ and *P*_sat_ are means of 2 (2011) or 3 (2010) shoots. The field fluorescence data corresponds to the average maximum nighttime quantum yield of PSII over 3–4 Monitoring PAM heads. The error bars denote standard deviation.

The initial slope of photosynthetic light response (α_f_) and light-saturated photosynthesis (*P*_sat_) followed similar seasonal courses over the time when both parameters could be estimated from the continuous gas exchange data taken in the field (Figure 2A). The field observations show that α_f_ stayed nearly as high as in the summer until early November. The data collected on the occasional sunny days in September and early October also reveal that the saturation of photosynthetic rate is shifted toward lower light in autumn when available radiation is low even on clear days. Clear-sky PPFD incident on a horizontal plane at noon drops below 500 μmol m^−2^ s^−1^ in October, after which the rate of light-saturated photosynthesis cannot be estimated from field data any more. However, the stronger saturation of the light response could be distinguished from field data until early November.

The monthly light response measurements in standard conditions confirmed the change in the light saturation of photosynthesis (results not shown). The initial slope α_s_ remained at summertime level until October and was relatively higher than the capacity of CO_2_ fixation (*P*_1200_) throughout the autumn (Figure 2B). The values of the light response parameters were at their lowest in December 2010 after a very cold spell and in February-March of 2010 and 2011. The end of year 2011 was mild (temperature did not drop below −6°C) and photosynthetic parameters in December were higher than in the end of the previous year, about one fourth of their mean summertime values. There was some potential for photosynthetic production in winter, which was not visible in the field measurements. *P*_1200_ in winter was typically in the order of 10% of its average value in June-August. At low light the relative potential (as indicated by the initial slope α_s_) could be even higher, up to 20% of summertime level (Figure 2B).

The operating quantum yield of fluorescence at 50 μmol m^−2^ s^−1^ PAR (Φ_PSII_, Figure 2C) and the initial slope of photosynthetic light response (α_s_, Figure 2B) showed similar seasonal patterns and were consistent with the seasonal pattern in the maximum quantum yield of PSII (*F*_v_/*F*_m_) estimated from the field monitoring data (Figure 2C). On the other hand, the high Φ_PSII_ or *F*_v_/*F*_m_ in autumn did not correspond to high photosynthetic capacity (*P*_1200_ or *P*_sat_, Figure 2A).

**Figure 3:**
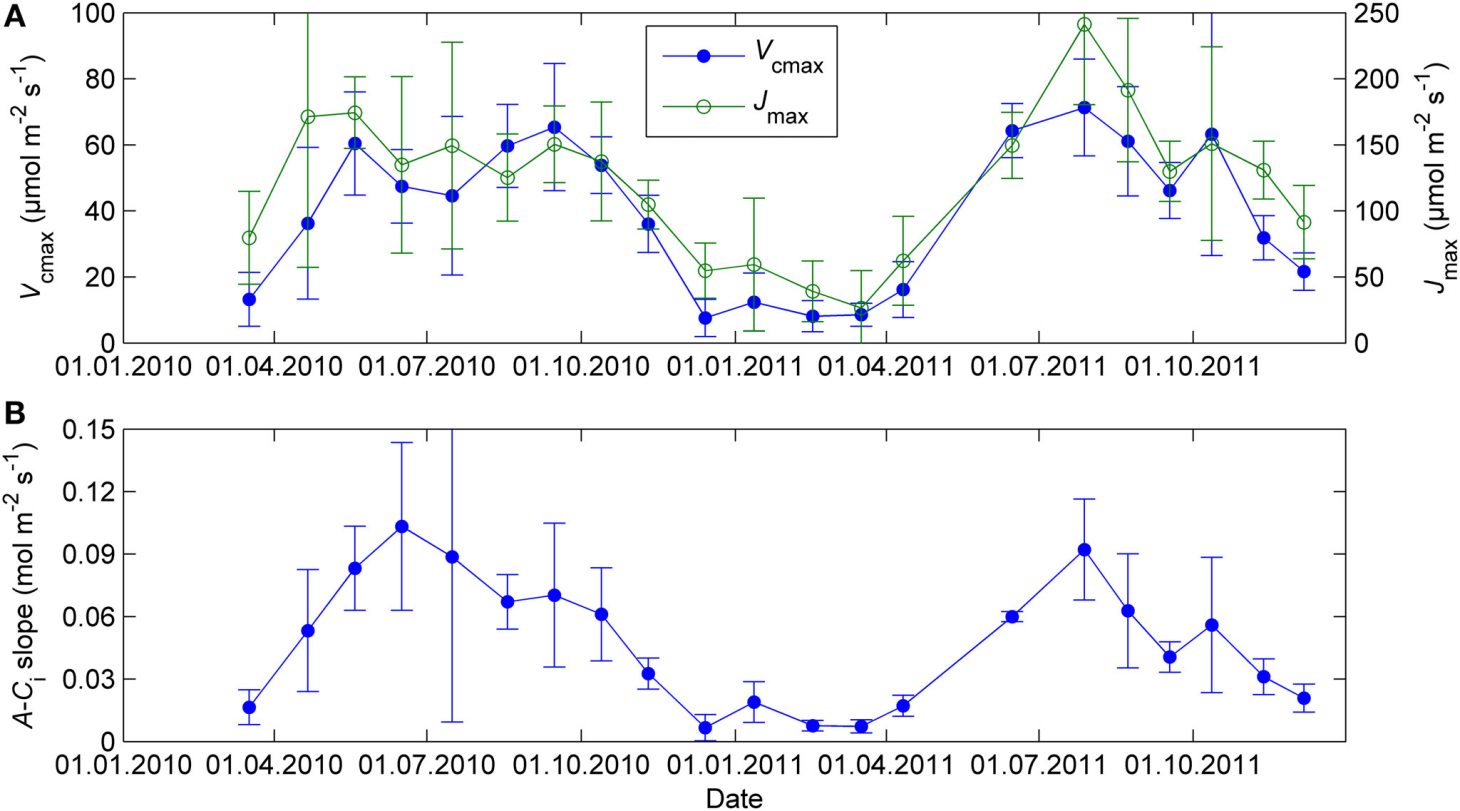
**Biochemical model parameters *V*_cmax_ and *J*_max_ (A) and the initial slope of *A*-*C*_i_ curve (B) from CO_2_ response measurements at 18°C and saturating light (1300 μmol m^−2^ s^−1^)**. The error bars denote standard deviation.

Biochemical model parameter related to the capacity of light reactions (*J*_max_) and the capacity of CO_2_ fixation (*V*_cmax_) showed little differentiation in their seasonal courses compared to the simple light response parameters (Figure 3). The seasonal course of *A*-*C*_i_ slope was slightly different from *V*_cmax_ but still inconsistent with *P*_1200_ and α_s_.

**Figure 4:**
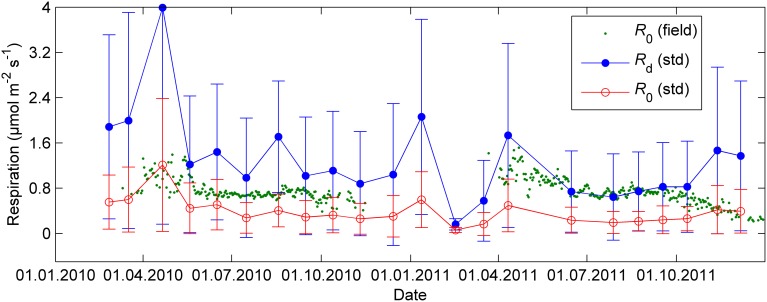
**Respiration in 18°C (*R*_d_), exponential relationship (Equation 10 with *Q*_10_ = 2) normalized to 0°C (*R*_0_, std) and *R*_0_ estimated from the automated cuvettes in the field (*R*_0_,field) during nights when air temperature was above 0°C**. The error bars denote standard deviation.

The continuous field measurements indicated that the base level of respiration *R*_0_, that is, respiration at 0°C, was higher in spring than in summer (Figure 4). The monthly response measurements largely agreed with the continuous data; in both study years *R*_0_ peaked in early spring, remained stable from June until September and declined again from late September on. Respiration as well as all photosynthetic parameters at 18°C were near zero in the February 2011 campaign after period of very low temperatures (*T*_min_ = −21°C, *T*_min_ of preceding 5 days = −24°C, Figures 2–4).

### Relationship between temperature and photosynthetic parameters

**Figure 5:**
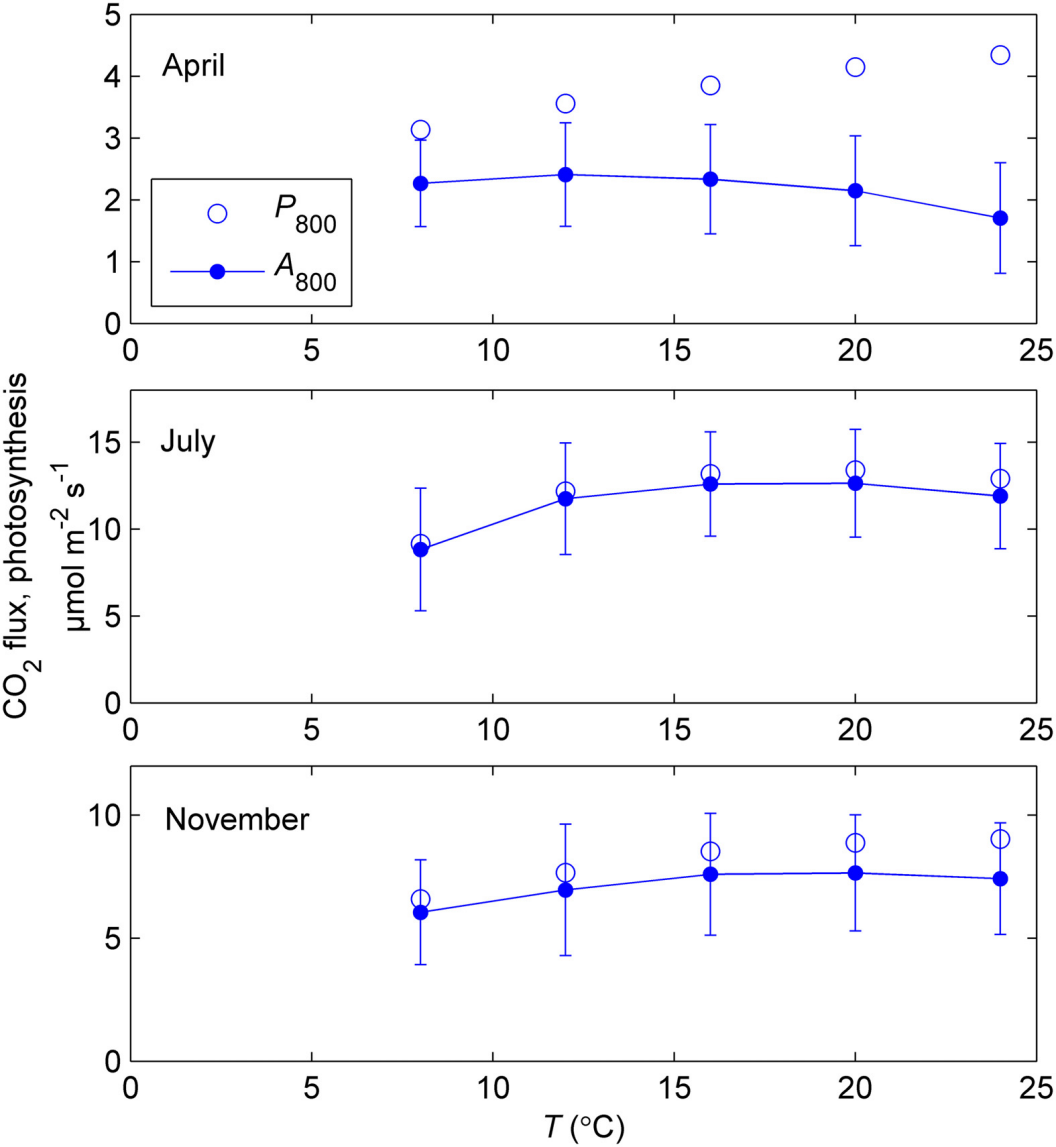
**Mean temperature responses of measured net CO_2_ exchange at 800 μmol m^−2^ s^−1^ (*A*_800_) and estimated photosynthesis (*P*_800_) of 5–6 sample shoots during response measurements in controlled conditions in spring (9–12 April), summer (18–26 July) and late autumn (11–14 November) of year 2011**. Photosynthesis was estimated from the net CO_2_ exchange by adding respiration measured in the dark. The error bars indicate standard deviation of net CO_2_ exchange. Note the different scales of vertical axes.

The temperature responses of net CO_2_ exchange at 800 μmol m^−2^ s^−1^ PAR (*A*_800_) determined during three response campaigns in 2011 were relatively flat but the optimum was at a lower temperature (12°C) in April than in July and November (16–20°C). When gross photosynthesis (*P*_800_) was estimated by adding the measured *R*_d_ to the net CO_2_ exchange, the temperature responses became similar in shape (Figure 5). When photosynthetic capacity is downregulated, estimation of the temperature response of photosynthesis is very sensitive to respiration.

**Figure 6:**
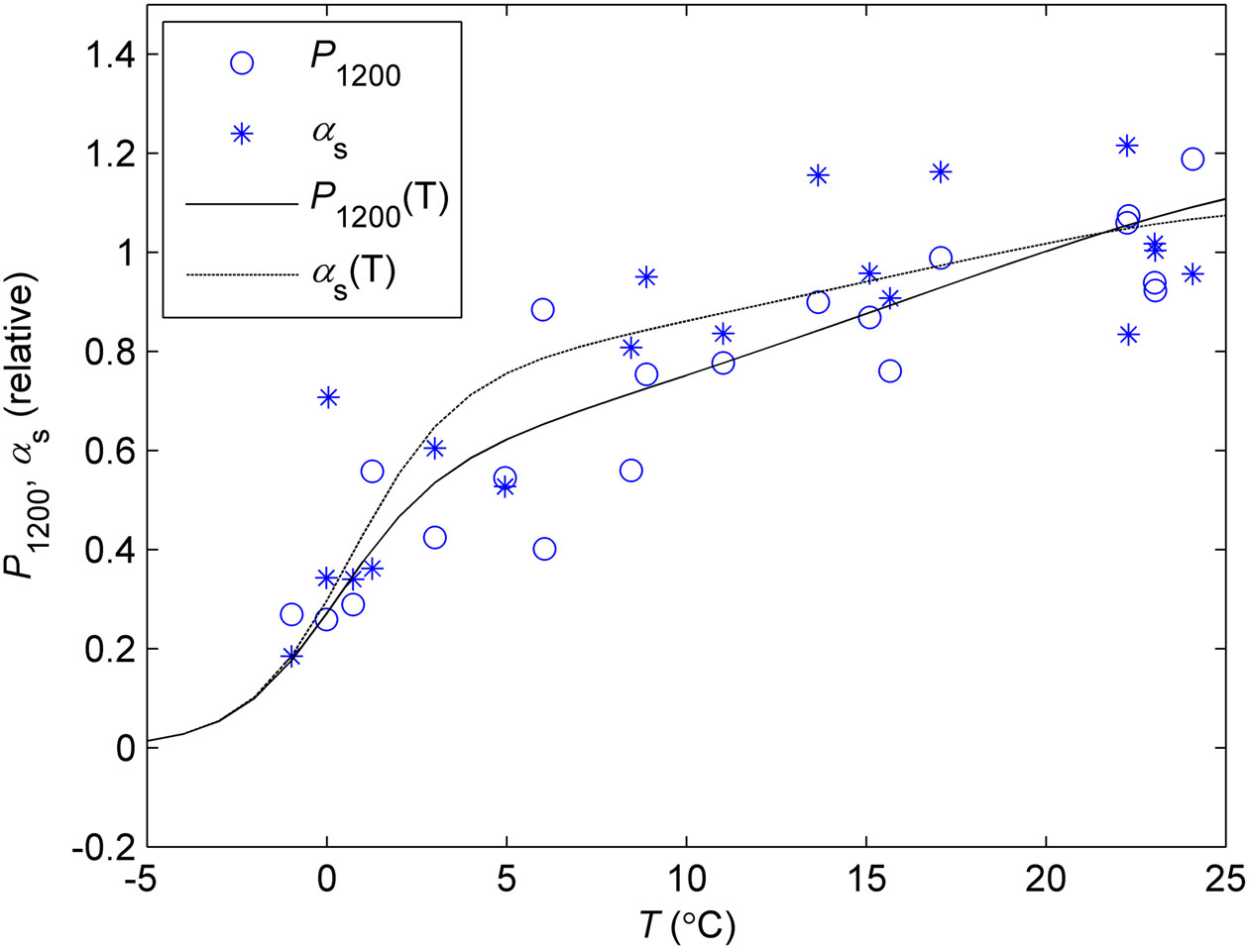
**Temperature response of *P*_1200_ and α_s_ determined from the light response measurements in controlled conditions**. Each symbol corresponds to the monthly value of *P*_1200_ or **α**_s_ determined in prevailing ambient temperature vs. corresponding parameter in the standard temperature (18°C). The lines denote the photosynthetic temperature response model (Equation 7) fitted to *P*_1200_ (*P*(T), solid line) and **α**_s_ (**α**(T), dash line). The parameters defining the the response at sub-zero temperatures were fixed to values that make the curve reach zero at −5°C based on the continuous chamber data from several years.

The initial slope of the light response (α_s_) and *P*_1200_ in the monthly response measurements showed similar relationships between the prevailing ambient temperature and reference temperature (Figure 6). Relative *P*_1200_ had slightly steeper slope of the temperature response than α_s_. Temperatures below zero were not used in the response measurements but the automatic chamber data indicates steep drop in photosynthetic rate below 0°C and the CO_2_ exchange signal, including respiration, diminishes at about −5°C (data not shown, see model approximation in Figure 6).

**Figure 7:**
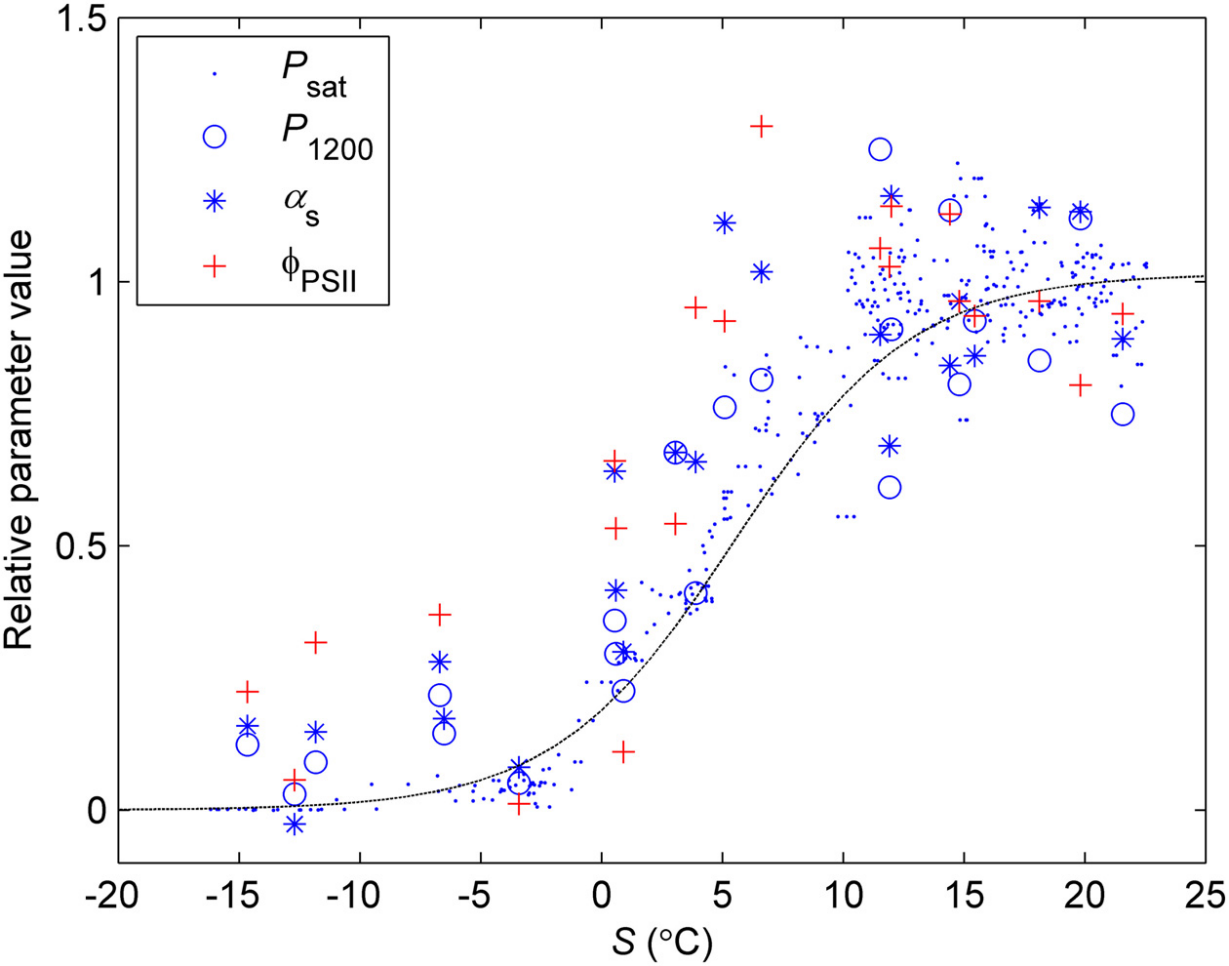
**Normalized values of light response parameters vs. state of acclimation *S* (Equation 8, time constant = 7 days)**. *P*_sat_ was estimated in ambient temperature from continuous chamber measurements, *P*_1200_ and **α**_s_ from light response measurements in controlled conditions and standard temperature (18°C). Dash line denotes the sigmoid relationship between *P*_sat_/*C*_i_ and *S* from Kolari et al. (2007). The parameter values are relative to the mean value of the respective parameter when 5-day mean temperature >15°C.

**Figure 8:**
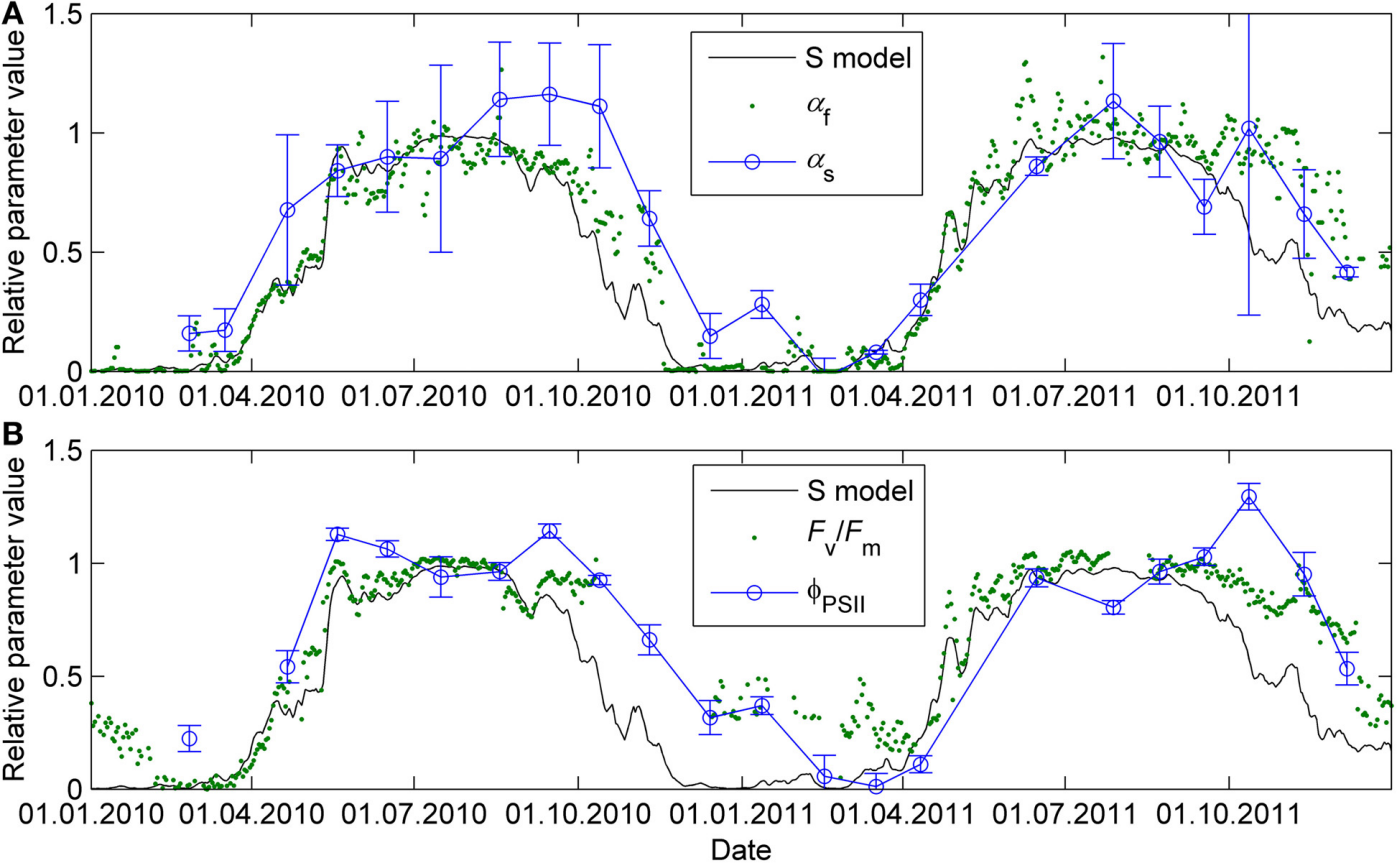
**Seasonal courses of relative photosynthetic parameters α_f_ and α_s_ (A) and *F*_v_/*F*_m_ and Φ_PSII_ (B) prediction with sigmoid response to the state of acclimation *S* (Equation 8 and 9, time constant τ = 7 days)**. The *S* prediction and parameter values were normalized to interval 0–1 where 0 represents the minimum value of the parameter and 1 the maximum. For parameters determined in standard conditions (α_s_, Φ_PSII_), the maximum is the mean parameter value of the respective parameter when 5-day mean air temperature was >15°C.

**Figure 9:**
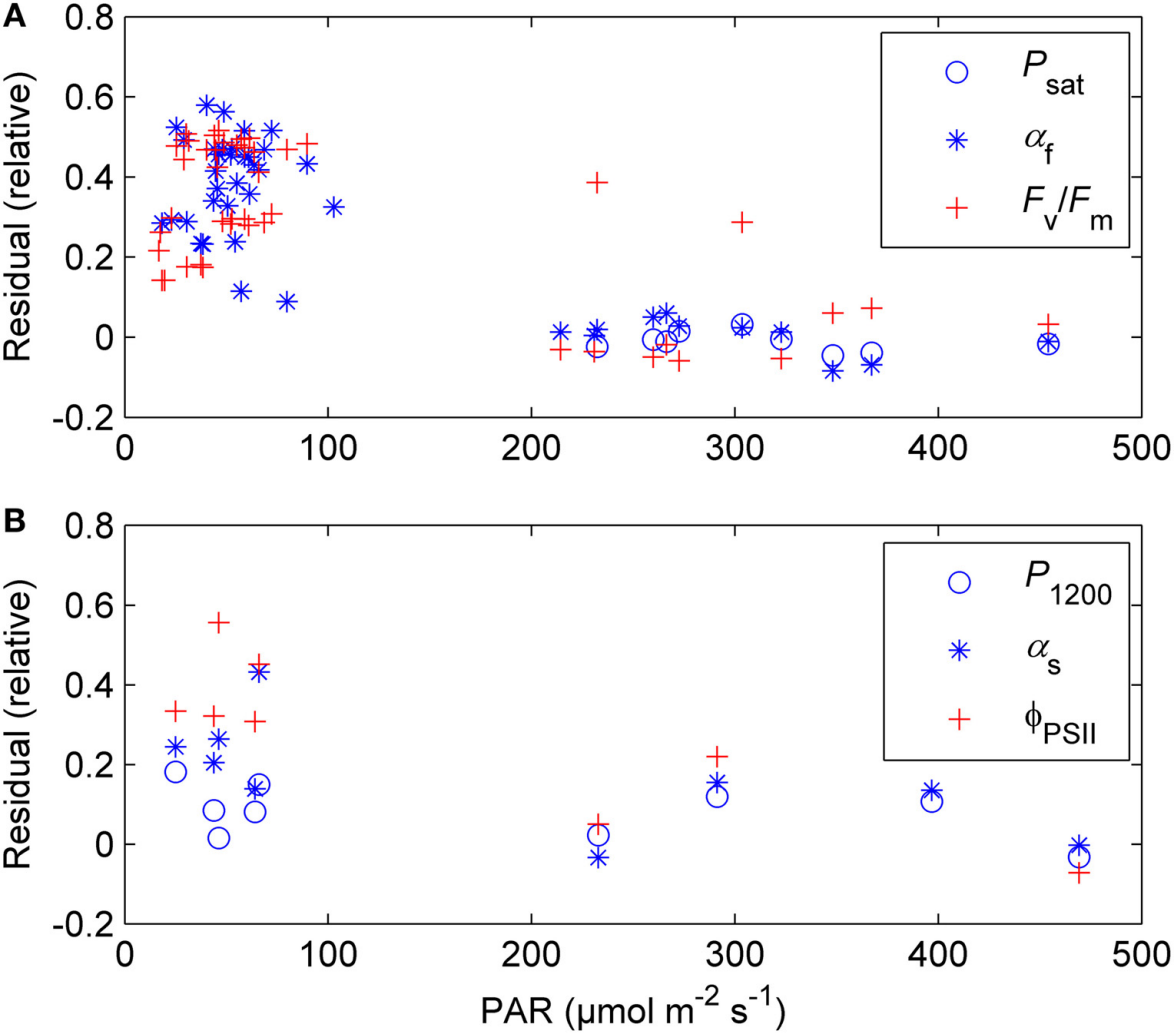
**The difference between normalized *P*_sat_, α_f_, and *F*_v_/*F*_m_ (A) and *P*_1200_, α_s_, and Φ_PSII_ (B) and the sigmoid *S* model prediction (Equation 9) as a function of prevailing light conditions (average PAR from sunrise to 12:00 during the day in question and two previous days)**. The plot comprises days from December through March when daytime (8:00–17:00 local normal time) air temperature was >0°C.

The previously reported sigmoid relationship between photosynthetic capacity parameter β in the optimal stomatal control model (Hari et al., 1986) and state of acclimation *S* (Kolari et al., 2007) agreed well with the field and the standard temperature parameters in spring (Figures 7, 8). On the other hand, light reaction parameters α_s_ and Φ_PSII_ in standard conditions were lagging the temperature-based prediction in autumn (Figure 8). In both study years the light response parameter values were higher in November than in March although prevailing temperatures were comparable. Furthermore, *F*_v_/*F*_m_ in the field was at the same level in December 2010-January 2011 and December 2011 although the latter period was warmer (Figure 1B) and preceded by mild weather. The lowest temperature of autumn 2011 was −6°C whereas in 2010 temperatures near −20°C were experienced already in late November. *F*_v_/*F*_m_ and Φ_PSII_ also continued slow decline until March 2011 and showed only weak signs of recovery in April despite the increasing trend in temperature. The difference between normalized α_s_, α_f_, Φ_PSII_ and *F*_v_/*F*_m_ and the *S* model prediction was negatively correlated with mean PAR during previous mornings (Figure 9) which suggests that the downregulation during winter is related to light environment rather than driven by temperature alone. The difference between spring and autumn could also be related to minimum temperatures. *P*_1200_ and α_s_ at 18°C were always <20% of their respective mean summertime levels when the minimum temperature of previous 24 h was −5°C and >20% when the minimum temperature was >−5°C (not shown).

## Discussion

### Seasonality of photosynthesis and respiration

The response measurements in standard conditions indicated a strong downregulation in the photosynthetic machinery toward winter and a recovery in spring. The seasonal patterns of the light response parameters and fluorescence were qualitatively similar to those determined from the continuous field measurements and in line with previous studies at the site (Kolari et al., 2007; Porcar-Castell et al., 2008a; Porcar-Castell, 2011). However, there were some notable differences. First, the response measurements revealed considerable potential for photosynthetic production most of the time in winter, which was not visible in the field measurements. Second, parameters related to light reactions (α_s_, Φ_PSII_) remained at nearly summertime level in autumn while carbon reaction-related parameter *P*_1200_ declined steadily from August to December.

Photosynthetic parameters obtained in standard conditions in winter were not as close to zero as apparent from the field data, but remained as high as 20% of the respective summertime values. This implies that about 80% of the reduction in momentary photosynthetic rates from summer to winter is attributed to slow changes in the capacity of light and carbon reactions. The rest is due to instantaneous responses that determine how much of the capacity is realized. This finding suggests that boreal evergreen forests would be able to rapidly capitalize from early warm spells. Indeed, wintertime photosynthesis has been observed in various studies on boreal winter-acclimated trees when temperature has been high enough (e.g., Ensminger et al., 2004; Sevanto et al., 2006). Exceptionally, all photosynthetic parameters as well as respiration at 18°C were near zero in the February 2011 campaign which took place immediately after a period of very low temperatures (minimum temperature of previous 5 days was −24°C).

The quantum yield of fluorescence (Φ_PSII_, *F*_v_/*F*_m_) and the initial slope of photosynthetic light response (α_s_, α_f_) had similar seasonal patterns in standard conditions and in the field. Representativeness of fluorescence as indicator of light-saturated photosynthetic capacity was much poorer: the high Φ_PSII_ in autumn did not correspond to high *P*_1200_. On the other hand, in spring the parameter values increased in concert in the field and in the standard conditions (Figure 2). Concluding the photosynthetic capacity from chlorophyll fluorescence overestimates the maximum capacity in autumn and early winter. Therefore, it is advisable to address light and carbon reactions separately in modeling and interpretation of empirical data, not only capacity (*J*_max_, *V*_cmax_, *P*_sat_) but also efficiency (α).

The shape of the photosynthetic temperature response in saturating light was similar in spring, summer and autumn although the absolute level varied considerably (Figure 5). In some modeling approaches temperature acclimation shifts the maximum of the response function toward lower temperature (e.g., Kattge and Knorr, 2007). In this study, such approach was not applicable. The response of photosynthetic rate to subzero temperature also calls for improvement to frequently used exponential-like temperature response models to address low-temperature inhibition, for instance, by including an additional term (Collatz et al., 1992, Equation 7). The steep drop in photosynthetic rate below 0°C and the diminishing CO_2_ exchange signal, including respiration, at about −5°C could be linked with extracellular freezing in the shoots, which takes place few degrees below zero (Brown et al., 1974; Lintunen et al., 2014). Freezing results in the decrease in leaf water potential which inhibits cell metabolism and may cause embolism in water-conducting tissues.

Biochemical photosynthetic model parameters *J*_max_ and *V*_cmax_ or *A*-*C*_i_ slope showed a strong seasonal variability in the response measurements. Unlike α_s_, Φ_PSII_, and *P*_1200_, the biochemical model parameters related to light and carbon reactions followed similar seasonal courses. However, one should be cautious when planning and interpreting *A*-*C*_i_ response measurements on winter-acclimated leaves; the usual assumptions of light and carboxylation limitations at different internal CO_2_ concentrations might not hold. Furthermore, light and carbon limitations are hard to separate from *A*-*C*_i_ data when the signal is small. A good practice would be to make also light response measurements and analyze them using simple light response models that are more robust with low signal and field data, as presented in this study.

Respiration also showed seasonal acclimation although not as conspicuous as photosynthesis. The field and response measurements were consistent in this parameter except in late autumn 2011 when respiration in the field declined whereas the response measurements showed relatively high rate of respiration. Peak values in the base level of respiration *R*_0_ were observed in April, too early to be directly linked with visible growth (Kolari et al., 2009), but they could reflect the biochemical processes related to recovery from winter dormancy and starting of growth. The autumn decline in *R*_0_ was consistent with the overall decrease in primary productivity: downregulated photosynthetic machinery requires little energy to maintain the low capacity. Considering the seasonality in the base level of respiration is also important when estimating photosynthetic parameters from field data. The level of respiration rate largely determines the observable CO_2_ assimilation rate and the shape of the apparent temperature response when the photosynthetic rate is low in winter and early spring.

### Environmental control of seasonal acclimation

The seasonality of photosynthetic capacity has been linked to prevailing temperature (Pelkonen and Hari, 1980; Mäkelä et al., 2004). The relationship between temperature and lumped photosynthetic capacity (β) via state of acclimation (*S*) was formulated by Mäkelä et al. (2004) and the concept further tested by, among others, Mäkelä et al. (2006) and Kolari et al. (2007). The sigmoid β-*S* relationship by Kolari et al. (2007) predicted well the photosynthetic parameters determined from continuous measurements in the field in spring (Figure 8). However, when the prevailing temperature was low, photosynthetic rate determined in the field underestimated the actual capacity in more favorable conditions. The temperature acclimation derived from the field measurements, thus, results from the instantaneous responses superimposed on the seasonal acclimation and overestimates the downregulation of the physiological state.

Temperature acclimation explained the recovery in spring better than the downregulation in autumn and winter (Figure 8). The parameters related to the efficiency of light reactions at low light (α, α_s_, Φ_PSII_, *F*_v_/*F*_m_) were considerably higher than the prediction based on temperature acclimation in autumn. Linkosalo et al. (2014) could explain the downregulation of fluorescence yield with temperature history using different model parameterizations for autumn and spring. However, the regulation mechanisms of light and carbon reactions are different, which creates a mismatch between e.g., Φ_PSII_ and *P*_sat_ during autumn. Light reactions are more sensitive to light but relatively insensitive to temperature. The difference in light-reaction parameter values between the field conditions and 18°C was small (Figures 2, 8) and the response measurements also indicated low instantaneous temperature sensitivity (Figure 6). Since boreal autumns are rather dark, there is little pressure to downregulate the light reactions (Ensminger et al., 2006; Busch et al., 2007) and the downregulation of the light harvesting machinery is not completed until late winter (Porcar-Castell et al., 2008a; Porcar-Castell, 2011). Carbon fixation is solely controlled by biochemical reactions, thus, it is more sensitive to temperature and the capacity also largely follows the prevailing temperature. Indirect regulation (sink limitation) may also contribute to the decline in photosynthetic rate and capacity in autumn. The different observed patterns in light and carbon limitation were consistent with the environmental conditions if we consider resource allocation in the photosynthetic machinery: low light in autumn is utilized efficiently whereas high maximum capacity is not needed and *P*_sat_ appears to be low. The ability of photosynthesize during warm spells, efficient photosynthesis at low light, and downregulated respiration are important for the trees to avoid excessive carbon loss during winter (Baldocchi, 2008; Piao et al., 2008; Vesala et al., 2010). On the other hand, the contribution of photoperiod to the photosynthetic capacity can limit the carbon uptake potential in warm winters (Bauerle et al., 2012).

We found that photosynthetic capacity in Scots pine varies seasonally much more than the base level of respiration (Figures 2–4). The strong seasonality of photosynthetic parameters in the boreal zone was also reported in many earlier studies (e.g., Leverenz and Öquist, 1987; Mäkelä et al., 2004). However, Gea-Izquierdo et al. (2010) found a decreasing trend in the magnitude and increase in the rate (smaller time constant τ) of photosynthetic acclimation across climatic gradient from northern boreal to temperate zone. In other words, the instantaneous temperature response dominates over slow temperature acclimation in the south. Ow et al. (2010), in turn, reported stronger acclimation in respiration than in photosynthesis for *Pinus radiata* at a site where lowest temperatures of the year were barely below 0°C. The relationship between photosynthetic capacity and temperature evidently depends on the range of seasonal variability in temperature, especially on the low end of the range, as apparent physiological activity ceases at about −5°C. Freezing could explain why the temperature acclimation is so much stronger in the north than in the south. Recovery from freezing-induced embolism as well as cold or frozen soil can bring additional slowness to the spring recovery of photosynthesis (Bergh et al., 1998).

## Conclusions

The seasonal patterns of photosynthesis and respiration result from instantaneous responses superimposed on seasonal acclimation. The measurements in controlled conditions revealed that notable residual photosynthetic capacity remained during winter especially in the light reactions of photosynthesis, which suggests that boreal trees are able to promptly utilize warm spells or milder winters in the changing climate. The base level of respiration also followed the decreasing trend in photosynthetic capacity in autumn.

The seasonal patterns of light and carbon reactions and respiration were different which implies that they should be considered separately. In addition to estimating capacity parameters (e.g., *J*_max_) it is important to consider the efficiency of light reactions (α). Chlorophyll fluorescence overestimated the level of light-saturated photosynthesis in autumn and early winter.

Slow temperature acclimation explained well the recovery of photosynthetic parameter values in spring whereas the decline of light reaction parameters in autumn and especially the final stages of the downregulation during winter could be linked with light rather than temperature. Minimum temperature might be another decisive factor in the seasonal acclimation of photosynthesis and it can explain the observed differences in the photosynthetic temperature acclimation between boreal and temperate trees. More attention should be paid to low-temperature responses in models. Furthermore, physiological responses in models describing soil-vegetation-atmosphere interactions are predominantly instantaneous responses (Smith and Dukes, 2013). Our results show that to be able to predict photosynthesis and respiration in more variable environmental conditions and more extreme seasonal dynamics, it is important to address both instantaneous responses and slow changes in the photosynthetic state. The importance is pronounced when considering climatic warming scenarios

### Conflict of interest statement

The authors declare that the research was conducted in the absence of any commercial or financial relationships that could be construed as a potential conflict of interest.

## References

[B1] Altimir N. Vesala T. Keronen P. Kulmala M. Hari P. (2002). Methodology for direct field measurements of ozone flux to foliage with shoot chambers. Atmos. Environ. 36, 19–29 10.1016/S1352-2310(01)00478-2

[B2] Baldocchi D. D. (2008). Breathing of the terrestrial biosphere: lessons learned from a global network of carbon dioxide flux measurement systems. Aust. J. Bot. 56, 1–26 10.1071/BT07151

[B3] Bauerle W. L. Oren R. Way D. A. Qian S. S. Stoy P. C.. (2012). Photoperiodic regulation of the seasonal pattern of photosynthetic capacity and the implications for carbon cycling. Proc. Natl. Acad. Sci. U.S.A. 109, 8612–8617. 10.1073/pnas.1119131109 22586103 PMC3365212

[B3a] Bergh J. McMurtrie R. E. Linder S. (1998). Climatic factors controlling the productivity of Norway spruce: a-model-based analysis. Forest Ecol. Manag. 110, 127–139 10.1016/S0378-1127(98)00280-1

[B4] Brown M. S. Pereira E. S. B. Finkle B. (1974). Freezing of nonwoody plant tissues. Plant Physiol. 53, 709–711. 10.1104/pp.53.5.709 16658774 PMC541430

[B5] Busch F. Hüner N. P. A. Ensminger I. (2007). Increased air temperature during simulated autumn conditions does not increase photosynthetic carbon gain but affects the dissipation of excess energy in seedlings of the evergreen conifer Jack Pine. Plant Physiol. 143, 1242–1253. 10.1104/pp.106.092312 17259287 PMC1820919

[B6] Collatz G. J. Ribas-Carbo M. Berry J. A. (1992). Coupled photosynthesis-stomatal conductance model for leaves of C4 plants. Aust. J. Plant Physiol. 19, 519–538 10.1071/PP9920519

[B7] Ensminger I. Busch F. Hüner N. P. A. (2006). Photostasis and cold acclimation: sensing low temperature through photosynthesis. Physiol. Plantarum 126, 28–44. 10.1111/j.1399-3054.2006.00627.x23230444

[B8] Ensminger I. Sveshnikov D. Campbell D. A. Funk C. Jansson S. Lloyd J. (2004). Intermittent low temperatures constrain spring recovery of photosynthesis in boreal Scots pine forests. Glob. Change Biol. 10, 995–1008 10.1111/j.1365-2486.2004.00781.x

[B9] Farquhar G. D. von Caemmerer S. Berry J. A. (1980). A biochemical model of photosynthetic CO_2_ assimilation in leaves of C3 species. Planta 149, 78. 10.1007/BF00386231 24306196

[B10] Gea-Izquierdo G. Mäkelä A. Margolis H. Bergeron Y. Black T. A. Dunn A.. (2010). Modeling acclimation of photosynthesis to temperature in evergreen conifer forests. New Phytol. 188, 175–186. 10.1111/j.1469-8137.2010.03367.x 20618918

[B11] Genty B. Briantais J.-M. Baker N. R. (1989). The relationship between the quantum yield of photosynthetic electron transport and quenching of chlorophyll fluorescence. Biochim. Biophys. Acta 990, 87–92 10.1016/S0304-4165(89)80016-9

[B12] Öquist G. Hüner N. P. A. (2003). Photosynthesis of overwintering evergreen plants. Annu. Rev. Plant Biol. 54, 329–355. 10.1146/annurev.arplant.54.072402.115741 14502994

[B13] Hari P. Keronen P. Bäck J. Altimir N. Linkosalo T. Pohja T. (1999). An improvement of the method for calibrating measurements of photosynthetic CO_2_ flux. Plant Cell Environ. 22, 1297–1301 10.1046/j.1365-3040.1999.00478.x

[B14] Hari P. Mäkelä A. Korpilahti E. Holmberg M. (1986). Optimal control of gas exchange. Tree Physiol. 2, 169–175. 10.1093/treephys/2.1-2-3.169 14975851

[B15] Kattge J. Knorr W. (2007). Temperature acclimation in a biochemical model of photosynthesis: a reanalysis of data from 36 species. Plant Cell Environ. 30, 1176–1190. 10.1111/j.1365-3040.2007.01690.x 17661754

[B16] Kitajima M. Butler W. L. (1975). Quenching of chlorophyll fluorescence and primary photochemistry in chloroplasts by dibromothymoquinone. Biochim. Biophys. Acta 376, 105–115. 10.1016/0005-2728(75)90209-1 1125215

[B17] Kolari P. Kulmala L. Pumpanen J. Launiainen S. Ilvesniemi H. Hari P. (2009). CO_2_ exchange and component CO_2_ fluxes of a boreal Scots pine forest. Boreal Environ Res. 14, 761–783.

[B18] Kolari P. Lappalainen H. K. Hänninen H. Hari P. (2007). Relationship between temperature and the seasonal course of photosynthesis in Scots pine at northern timberline and in southern boreal zone. Tellus B 59, 542–552 10.1111/j.1600-0889.2007.00262.x

[B19] Leverenz J. W. Öquist G. (1987). Quantum yields of photosynthesis at temperatures between –2°C and 35°C in cold-tolerant C3 plant (*Pinus sylvestris*) during the course of one year. Plant Cell Environ. 10, 287–295 10.1111/j.1365-3040.1987.tb01608.x

[B21] Linkosalo T. Heikkinen J. Pulkkinen P. Mäkipää R. (2014). Fluorescence measurements show stronger cold inhibition of photosynthetic light reactions in Scots pine compared to Norway spruce as well as during spring compared to autumn. Front. Plant Sci. 5:264. 10.3389/fpls.2014.00264 24982664 PMC4055857

[B22] Lintunen A. Lindfors L. Kolari P. Juurola E. Nikinmaa E. Hölttä T. (2014). CO_2_ bursts during freezing offer a new perspective on avoidance of winter embolism in trees. Ann. Bot. 114, 1711–1718. 10.1093/aob/mcu190 25252688 PMC4649691

[B23] Mäkelä A. Hari P. Berninger F. Hänninen H. Nikinmaa E. (2004). Acclimation of photosynthetic capacity in Scots pine to the annual cycle temperature. Tree Physiol. 24, 369–378. 10.1093/treephys/24.4.369 14757576

[B24] Mäkelä A. Kolari P. Karimäki J. Nikinmaa E. Perämäki M. Hari P. (2006). Modelling five years of weather-driven variation of GPP in a boreal forest. Agric. For. Meterol. 139, 382–398 10.1016/j.agrformet.2006.08.017

[B26] Maxwell K. Johnson G. N. (2000). Chlorophyll fluorescence—a practical guide. J. Exp. Bot. 51, 659–668. 10.1093/jexbot/51.345.659 10938857

[B27] Nikinmaa E. Hölttä T. Hari P. Kolari P. Mäkelä A. Sevanto S.. (2013). Assimilate transport in phloem sets conditions for leaf gas exchange. Plant Cell Environ. 36, 655–669. 10.1111/pce.12004 22934921

[B28] Ow L. F. Whitehead D. Walcroft A. S. Turnbull M. H. (2010). Seasonal variation in foliar carbon exchange in *Pinus radiata* and *Populus deltoides*: respiration acclimates fully to changes in temperature but photosynthesis does not. Glob. Change Biol. 16, 288–302 10.1111/j.1365-2486.2009.01892.x

[B29] Pearcy R. W. (1990). Sunflecks and photosynthesis in plant canopies. Annu. Rev. Plant Physiol. Plant Mol. Biol. 41, 421–453 10.1146/annurev.pp.41.060190.002225

[B30] Pelkonen P. Hari P. (1980). The dependence of the springtime recovery of CO_2_ uptake in Scots pine on temperature and internal factors. Flora 169, 398–404.

[B31] Pfannschmidt T. (2003). Chloroplast redox signals: how photosynthesis controls its own genes. Trends Plant Sci. 8, 33–41. 10.1016/S1360-1385(02)00005-5 12523998

[B31a] Piao S. L. Ciais P. Friedlingstein P. Peylin P. Reichstein M. Luyssaert S.. (2008). Net carbon dioxide losses of northern ecosystems in response to autumn warming. Nature 451, 49–52. 10.1038/nature06444 18172494

[B32] Pirinen P. Simola H. Aalto J. Kaukoranta J.-P. Karlsson P. Ruuhela R. (2012). Tilastoja Suomen ilmastosta 1981–2010 (Climatological statistics of Finland 1981–2010). Finn. Meteorol. Inst. Rep. 2012:1, 1–81.

[B33] Porcar-Castell A. (2011). A high-resolution portrait of the annual dynamics of photochemical and non-photochemical quenching in needles of *Pinus sylvestris*. Physiol. Plantarum 143, 139–153. 10.1111/j.1399-3054.2011.01488.x 21615415

[B34] Porcar-Castell A. Juurola E. Ensminger I. Berninger F. Hari P. Nikinmaa E. (2008a). Seasonal acclimation of photosystem II in *Pinus sylvestris*. II. Using the rate constants of sustained thermal energy dissipation and photochemistry to study the effect of the light environment. Tree Physiol. 28, 1483–1491. 10.1093/treephys/28.10.1483 18708330

[B35] Porcar-Castell A. Pfündel E. Korhonen J. F. J. Juurola E. (2008b). A new monitoring PAM fluorometer (MONI-PAM) to study the short and long-term acclimation of photosystem II in field conditions. Photosyn. Res. 96, 173–179. 10.1007/s11120-008-9292-3 18283558

[B36] Schreiber U. Schliwa U. Bilger W. (1986). Continuous recording of photochemical and non-photochemical chlorophyll fluorescence quenching with a new type of modulation fluorometer. Photosyn. Res. 10, 51–62. 10.1007/BF00024185 24435276

[B37] Sevanto S. Suni T. Pumpanen J. Grönholm T. Kolari P. Nikinmaa E.. (2006). Wintertime photosynthesis and water uptake in a boreal forest. Tree Physiol. 26, 749–757. 10.1093/treephys/26.6.749 16510390

[B37a] Smith N. G. Dukes J. S. (2013). Plant respiration and photosynthesis in global-scale models: incorporating acclimation to temperature and CO_2_. Glob. Change Biol. 19, 45–63. 10.1111/j.1365-2486.2012.02797.x 23504720

[B38] Tiren L. (1927). Om barrytans storlek hos tallbestånd. Meddelanden Från Statens Skogsförsöksanstalt 23, 295–336.

[B38a] Vesala T. Launiainen S. Kolari P. Pumpanen J. Sevanto S. Hari P. (2010). Autumn temperature and carbon balance of a boreal Scots pine forest in Southern Finland. Biogeosciences 7, 163–176 10.5194/bg-7-163-2010

[B39] Wullschleger S. D. (1993). Biochemical limitations to carbon assimilation in C3 plants – a retrospective analysis of the A/C_i_ curves from 109 species. J. Exp. Bot. 44, 907–920 10.1093/jxb/44.5.907

